# Editorial: Insights in adolescent and young adult psychiatry: 2023

**DOI:** 10.3389/fpsyt.2024.1446846

**Published:** 2024-08-13

**Authors:** David Cohen

**Affiliations:** Department of Child and Adolescent Psychiatry, CNRS UMR 7222 Institut des Systèmes Intelligents et Robotiques, GH Pitié-Salpêtrière, APHP, Sorbonne Universités, Paris, France

**Keywords:** depression, adolescence, young adult, epidemiology, suicidality, marijuana

The “Adolescent and young adult psychiatry” section of Frontiers in Psychiatry is young, but the needs in the field are enormous (1). The post-COVID-19 pandemic is reminding practitioners every day of the huge numbers of referrals to emergency rooms they have to deal with (2). The 2023 Insights that gather 'works from our Editorial Board members reflect several heterogeneous issues embracing the discipline at large. This is not surprising given the complexity of our discipline. I am pleased that accepted manuscripts come from many countries and cultures.

First, Laporte et al. from Canada describe the ethical dilemma many face when dealing with referrals for acute mental health issues in children and adolescents. They explore how the triage model, on one hand, and the crisis model, on the other, have been proposed to solve it within emergency rooms. They compare the two models and discuss their merits (Laporte et al.).

Second, four contributions are dealing with mood and anxiety disorders in teens. In a large representative sample, Song et al. illustrate how body characteristics (objective height and subjective perceived obesity) are associated with suicide ideation in Korean adolescents, the effect being more prominent in female teens (Song et al.). This study highlights the need for more studies exploring gender effects in juvenile psychiatry. In an elegant quantitative study, Qin et al. explored how parents dealing with teens who exhibit repeated non-suicidal self-injury, accumulate a wealth of experience during their long-term care. Specifically, the authors investigated both the motivation to share these experiences and the barriers to doing so (Qin et al.). Next, Huang et al. explored the mediating role of anxiety and depression on perceived stress and quality of sleep among medical students during the COVID-19 pandemic. They highlight the need for a preventive approach in this at-risk population. Medical students have been repeatedly identified as a vulnerable population to mood disorders and suicide (3). Finally, Chen et al. explored the association between domestic violence and cyberbullying behavior in school and evidenced the mediating role of depression in school students. Maltreatment and bullying for children and adolescents have been identified as key factors for increasing the risk of mood disorders. The current study investigates the complexity of children’s trajectories by underlining the possibility of being both victim and perpetrator to some extent (Chen et al.).

Third, the large Jamaican study on Marijuana use in teens confirmed in this unique sample that the main correlates are similar to those in other countries. They include individual psychosocial risks (e.g., loneliness, frequent worry, suicidal ideation, physical attacks, early initiation, and school absenteeism); and family factors (e.g., parental smoking, parental support). Similarly, to studies in other contexts, the study insists on the co-occurrence of poly consumption and at-risk behaviors (4, 5). They conclude with the need for holistic intervention and policies (Dadras).

The next two studies investigate original therapeutic interventions that usually receive less interest in clinical research. Winds et al. report how a professional photoshoot intervention affects self-esteem and emotions in girls and boys from child and adolescent psychiatry clinics differentially. This is an original proposal as the assessment of cultural mediation as a therapeutic intervention is limited in our field. Most studies are descriptive or qualitative (e.g., 6). Then, Meng et al. reported a promising pilot trial of systematic couple group therapy for families of depressed adolescents. This is encouraging as the family approaches that many professionals in the field consider central to common practice do not receive enough attention in research compared to individual psychotherapy (7).

We end this brief overview of the Research Topic with a study exploring the neurobiology of depression. The serotonin theory hypothesis of depression has been explored for decades. The last large umbrella review of the evidence has shown some deception. The main areas of serotonin research provide no consistent evidence of an association between serotonin and depression, and no support for the hypothesis that depression is caused by lowered serotonin activity or concentrations. Some evidence supports that long-term antidepressant use reduces serotonin concentration (8). In their report, Ilavská et al. investigated how kynurenine and serotonin pathways interact after supplementation with omega-3 fatty acids that may improve depressive symptoms in children (9). They found that omega-3 FAs stimulated both kynurenine (kynurenine/tryptophan ratio) and serotonin (5-hydroxytryptophan) pathways, whereas omega-6 FAs only increased the kynurenine/tryptophan ratio. The meaning in terms of pathophysiology and/or use as a biomarker needs to be explored more in depth to claim any relevance in clinical practice.
